# Nano-Silver Ink of High Conductivity and Low Sintering Temperature for Paper Electronics

**DOI:** 10.1186/s11671-019-3011-1

**Published:** 2019-06-06

**Authors:** Lixin Mo, Zhenxin Guo, Zhenguo Wang, Li Yang, Yi Fang, Zhiqing Xin, Xiu Li, Yinjie Chen, Meijuan Cao, Qingqing Zhang, Luhai Li

**Affiliations:** 1 Beijing Engineering Research Center of Printed Electronics, Beijing Institute of Graphic Communication, Beijing, 102600 People’s Republic of China; 2 Research Institutes of Sweden (RISE), RISE Bioeconomy, Drottning Kristinas väg 61, 11428 Stockholm, Sweden

**Keywords:** Flexible and printed electronics, Paper electronics, Nano-silver, Low sintering temperature

## Abstract

Highly conductive ink with low sintering temperature is important for printed electronics on paper substrate. Silver nanoparticles (Ag NPs) of different average radii ranging from 48 to 176 nm were synthesized by adjusting the Ag^+^ concentration in the reaction process. The electric resistivity of the Ag NP-based ink film in relation to Ag NP size, sintering temperature, amount of PVP capping agent on Ag NP surface, and morphology evolution of the film during heating process was investigated. It was found that the resistivity of the films reduced very rapidly with increasing particle size due above all to reduced amount of protective agent capping on the Ag NPs. A semi-empirical relationship between the resistivity and the particle size was proposed. With the help of this mathematical expression, one gains both systematic and detailed insight to the resistivity evaluation with regard to the Ag particle size. The optimal electric resistivity of 4.6 μΩ cm was achieved at 140 °C for 10 min which was very close to the resistivity value of bulk Ag (1.58 μΩ cm). Mechanical flexibility of the printed electronics with the Ag NP-based ink on paper substrates was investigated. The prints on the art coated paper exhibited better flexibility compared to that on the photopaper. This might be attributed to the surface coating composition, surface morphology of the paper, and their corresponding ink absorption property.

**Electronic supplementary material:**

The online version of this article (10.1186/s11671-019-3011-1) contains supplementary material, which is available to authorized users.

## Introduction

Paper-based electronics has attracted great interests of research as it offers many irreplaceable superiorities [1–6]. Not only is paper widely available and inexpensive, it is also lightweight, biodegradable, and super flexible, which makes it a promising substrate for various electronics including flexible solar cells, displays, radio frequency identification (RFID) tags, thin film transistors, touch pads, and energy-storage devices [7–16]. Printed electronics on paper substrates has been considered as the major enabler of intelligent packaging functionalities, e.g., in tracing and tracking, gods and storage management, logistics and transport, and anti-counterfeiting. According to IDTechEx, the market demands were projected to be over $1.45 billion in year 2024 [17].

Requirements on high sintering temperature of metallic nanoparticle-based ink have been a limiting factor for paper-based printed electronics, as paper substrate may undergo dimensional changes during sintering process which cause de-laminations, cracking, etc. [18, 19]. Thus, the high conductivity and low sintering temperature have been the focus of the research. Magdassi et al. [20], Grouchko et al. [21], and Tang et al. [22] realized room temperature sintering of the Ag NPs by adding the destabilizing agents, oppositely charged polyelectrolytes, and Cl^−^ containing electrolyte, respectively, into the ink to promote the NP aggregation and coalescence in the drying processes. The optimized electric conductivities achieved were 20%, 41%, and 16%, respectively, of that of bulk silver. Both Xu et al. [23] and Wang et al. [24] introduced pressure into the Ag NP hot sintering process to decrease the heating temperature. It was found that the pressure could facilitate a more uniform and denser film microstructure, resulting in higher conductivity at relative low temperature. The electric resistivities obtained at 120 °C was 14.3 μΩ cm while with pressure of 25 MPa it reduced to 3.92 μΩ cm. In addition, some other sintering methods [25] have been involved to improve the metallic NP sintering at mild heating conditions such as photonic sintering [26–32], plasma [33–35], and microwaves [36, 37]. However, these methods either required electrolyte addition into the ink formulation which might deteriorate the stability of the metallic NP-based ink or expansive equipment and high energy consumption. Therefore, there is an unmet need for the metallic conductive ink that possesses high electric conductivity at relative low sintering temperature without involving either complex treatment or expensive equipment. An alternative approach is a chemical reaction, wherein the metallic source is either a molecular precursor or cation [38, 39]. By optimizing the molecular structure and the ink components, it was possible to deposit and to form conductive metal film at low temperature. However, the relatively low metal content and low viscosity limited its application in paper-based electronics.

We propose a new approach for obtaining Ag NP-based inks of high conductivity and low sintering temperature. The relationship between the electric conductivity of the ink film and the major influencing factors, e.g., Ag NP size, sintering temperature, amount of PVP protective agent, and film morphology, was studied. The mechanical flexibility of the printed electronics on paper substrates was also investigated.

## Methods

### Materials

Polyvinyl pyrrolidone (PVP, K30, MW = 58,000), ethylene glycol (EG), silver nitrate (AgNO_3_), and hydrazine hydrate (N_2_H_4_·H_2_O) were purchased from Aldrich (St. Louis, MO, USA). Acetone, isopropanol, and 2-butoxy ethanol were obtained from Beijing Chemical Works (Beijing, China). All chemical reagents were analytically pure, and no further purification was made.

### Synthesis and Characterization of the Ag NPs and Coated Films

The Ag NPs were synthesized by phase reduction method. In brief, 100 mL AgNO_3_ solution (1 g/mL) and 60 mL N_2_H_4_·H_2_O solution (0.8 g/mL, as reductant) were added drop-wise into 600 mL PVP solution (0.03 g/mL) that served as a protective agent at 10 °C. After 0.5 h reaction, Ag NPs were obtained by adding sufficient amount of acetone into the yellow-brownish Ag NP suspension so that Ag NPs were precipitated. Then, the Ag NP paste was re-dispersed into D.I. water again followed by acetone sedimentation. Such a process noted as washing in the upcoming sections was repeated a number of times to reduce the absorbed PVP on the surface of Ag NPs and to get desired concentration. The Ag NPs in different sizes and distributions, marked as S1 to S4 hereafter, were obtained by adjusting the reaction concentrations of the Ag^+^ with 0.385 mol L^−1^, 0.770 mol L^−1^, 1.540 mol L^−1^, and 1.925 mol L^−1^, respectively.

The X-ray diffraction (XRD) patterns of the Ag NPs with different reaction concentrations of Ag^+^ were characterized on the X-ray diffractometer (Rigaku Miniflex 600) with Cu Kα radiation operated at 40 kV, 15 mA, and a scanning rate of 5° min^−1^. The morphology and size distribution of the Ag NPs were obtained by scanning electron microscopy (SEM, Nanosem 430). Thermogravimetric analysis (TGA) profiles of the Ag NP concentrations with respect to different particle sizes and washing times were obtained by TA Instrument TGA-Q500 under N_2_ atmosphere in a heating rate of 10 °C/min. The Ag NPs were then spin-coated onto the glass slides, followed by heating on a hot plate in ambient environment at different temperatures from 30 to 140 °C for 10 min. The electrical resistivity of the coated film (Ag NP film) was calculated from sheet resistance and film’s thickness, measured with RTS-9 four-point probe station and the SEM, respectively.

### Preparation of the Ag NP-Based Inks and Characterization of Their Mechanical Flexibility on Paper Substrates

The Ag NP-based conductive inks for direct writing and screen printing were formulated by adding the concentrated Ag NP paste into a certain amount of EG, isopropanol, and 2-butoxy ethanol mixture (2:1:1 in volume) with the load of 20 wt.% and 70 wt.%, respectively. The direct writing Ag NP-based ink was filled into the ordinary commercial mark pen to make a conductive mark pen.

The mechanical flexibility of the Ag NP-based ink on paper was investigated. First, the linear arrays of 5 silver electrodes were drawn by the conductive mark pen on art coated paper and photopaper respectively following by 120 °C heating for 10 min. The dimensions of the silver electrode arrays were 60 mm in length, 7 mm in width, and 10 mm in spacing. Then, the testing samples on different paper were folded to the following bend radii, 2.5 mm, 1.0 mm, and 0.5 mm respectively, in 1000 cycles. We measured the electrical resistance change rate, (*R* − *R*_0_)/*R*_0_, as a function of bend radius and number of bend cycles, wherein the average electrical resistance values were obtained from the 5 silver electrodes.

### Applications of the Ag NP-Based Ink for Paper Electronics

A 7-segment digital display circuit was directly hand drawn using the conductive mark pen on art coated paper. Meanwhile, a high-frequency RFID antenna was screen printed on the art coated paper. Both of the paper-based electric devices were treated at 120 °C for 10 min.

## Results and Discussion

### Characteristics of the Synthesized Ag NPs Using Various Ag^+^ Concentrations

**Fig. 1:**
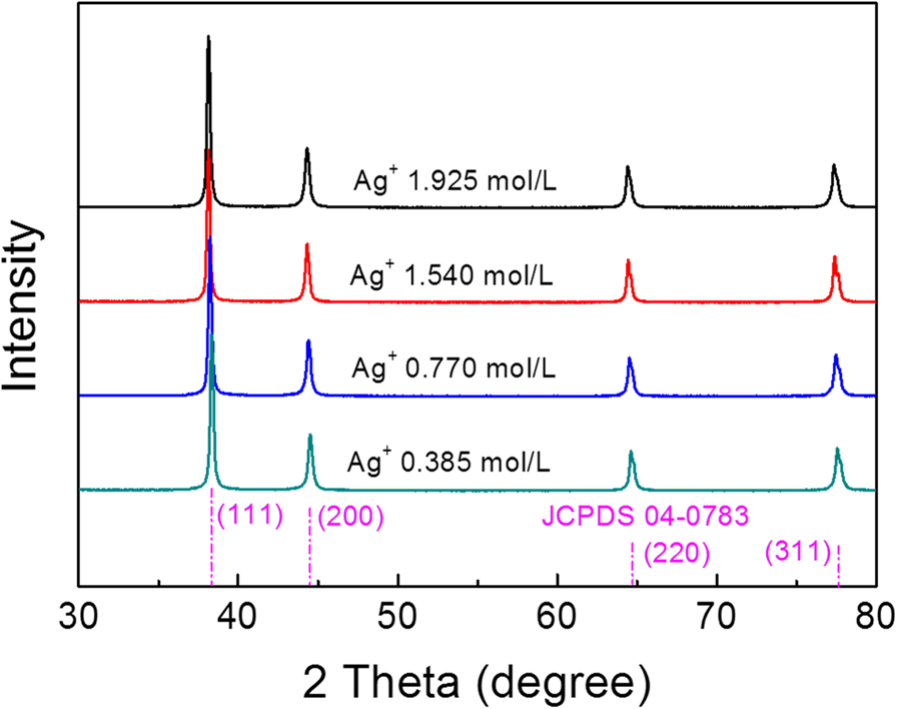
XRD patterns of Ag NPs synthesized using different Ag^+^ concentrations. The corresponding Ag^+^ concentrations of 0.385 mol L^−1^, 0.770 mol L^−1^, 1.540 mol L^−1^, and 1.925 mol L^−1^ were indicated in the figure. The reference patterns of silver (JCPDS 04-0783) was also shown

**Fig. 2:**
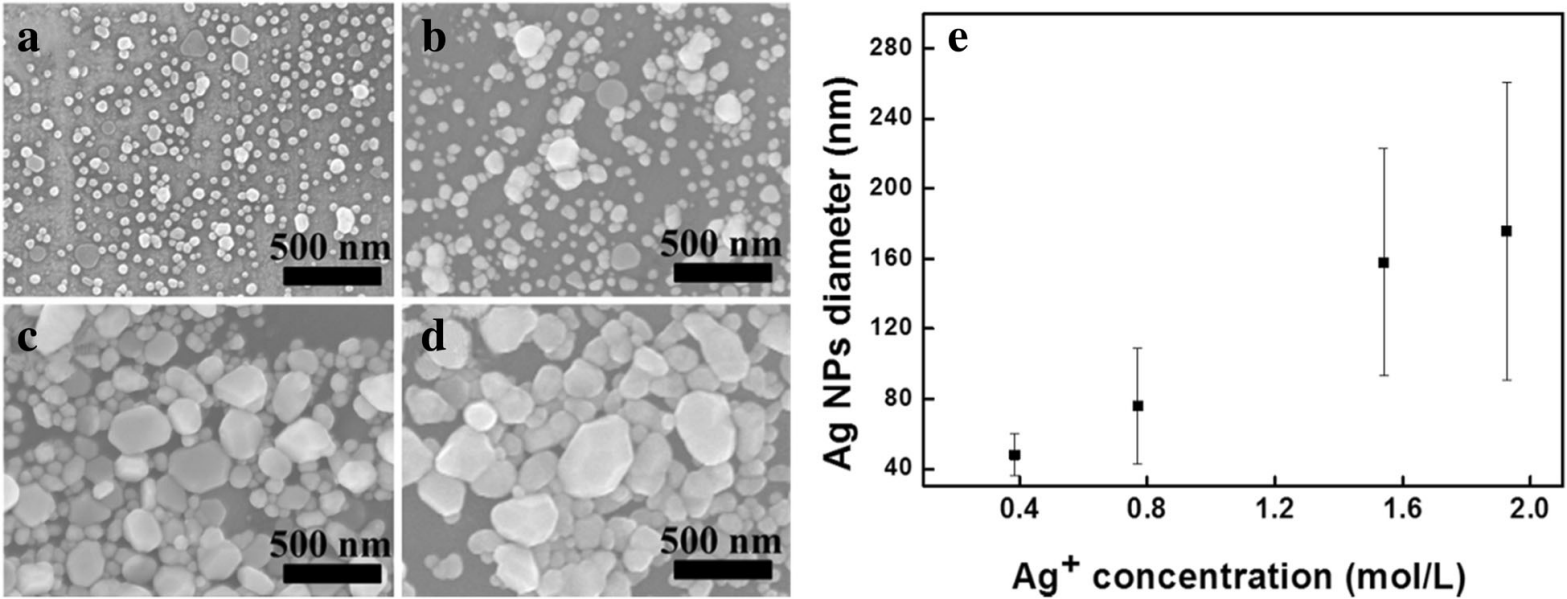
SEM images of the synthesized Ag NPs with different size distributions by adjusting the Ag^+^ concentration in reaction. **a** Ag^+^ 0.385 mol L^−1^. **b** Ag^+^ 0.770 mol L^−1^. **c** Ag^+^ 1.540 mol L^−1^. **d** Ag^+^ 1.925 mol L^−1^. **e** Average diameter of Ag NPs with respect to the Ag^+^ concentration

Figure 1 shows the XRD patterns of the Ag NPs synthesized using various Ag^+^ concentrations in the reaction. These XRD patterns exhibited only the peaks of metallic silver (JCPDS 04-0783) without any other signals, indicating that the synthesized samples are highly purified and face-centered cubic (fcc)-phased Ag NPs. The fact that no surface oxide observed in Ag NPs is important, because silver oxides have much lower electric conductivity and might prevent the sintering of Ag NPs at relatively low temperature. The SEM images of the synthesized Ag NPs using different Ag^+^ concentrations in the reaction solutions are shown in Fig. 2a–d. The Ag NPs with diameters of 48 ± 12 nm, 76 ± 33 nm, 158 ± 65 nm, and 176 ± 85 nm were obtained from the Ag^+^ concentrations of 0.385 mol L^−1^, 0.770 mol L^−1^, 1.540 mol L^−1^, and 1.925 mol L^−1^, respectively, denoted as S1, S2, S3, and S4. The change in the average diameters of the synthesized Ag NPs in relation to the Ag^+^ concentration used is shown in Fig. 2e. The average size of Ag NPs increased from 48 to 176 nm, and their size distributions also became broader with the increasing Ag^+^ concentrations. This was attributed to two reasons. First, a higher Ag^+^ concentration means longer feeding time of the AgNO_3_ solution in the reaction solution, hence extended growth time of the Ag NPs. On the other hand, the relative small amount of the protective agent PVP compared to the increasing Ag^+^ concentration could not prevent the growth and aggregation of Ag NPs more effectively, leading to formation of Ag NPs of bigger sizes. This result suggested that adjusting the Ag^+^ concentration helped control the size of Ag NPs over a relatively wide range.

### The Amount of PVP Capping on the Surface of Ag NPs

It is well known that the insulating PVP capping on the surfaces of the silver nanoparticles reduced the electron mobility within the Ag NP film, leading to significantly decreased conductivity. Thus, the amount of PVP capping on the surface of silver nanoparticles has to be reduced in order to enhance its conductivity at relatively low temperature. This can be achieved by the washing process described in the “Methods” section.

#### The Effect of Wash Times on the Amount of PVP

**Fig. 3:**
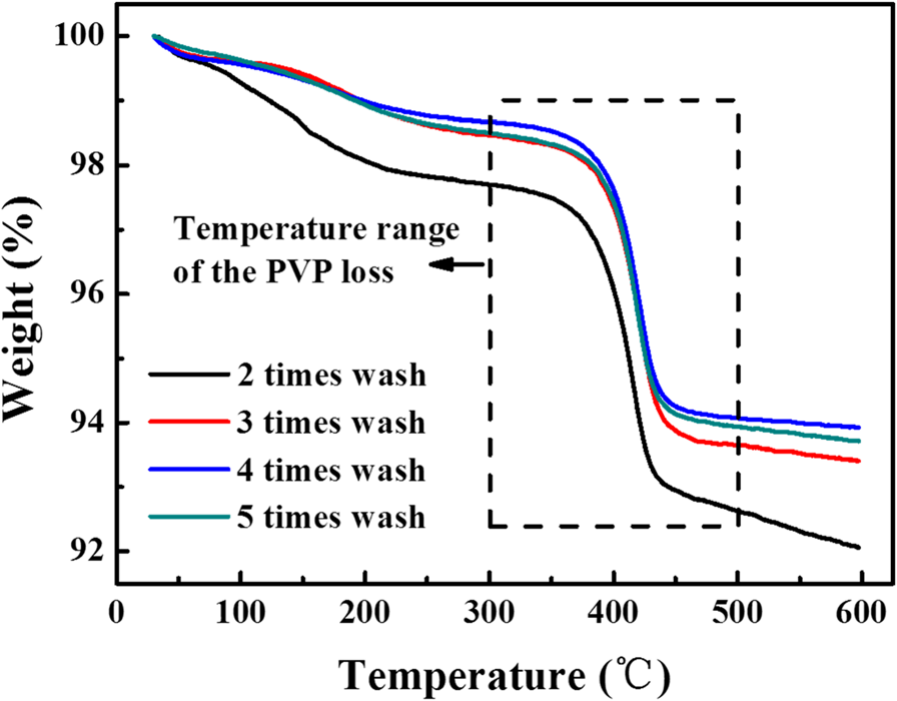
TGA curves of the Ag NP suspensions of S1 with respect to washing times

Figure 3 shows the TGA curves of the Ag NP suspensions of S1 after being washed for two to five times. These four TGA curves exhibit a similar temperature dependence profile. In each of the wash processes, the continuous loss of weight from the initial temperature to about 300 °C can be attributed to the evaporation of the solvents. Another significant weight loss was observed in the temperature range between 300 and 500 °C, marked by the dashed-line rectangle box. This temperature range overlaps with the decomposition temperature range of PVP, causing desorption and decomposition of the PVP from the surface of the Ag NPs. The residual mass at relatively high temperature of 600 °C represents the silver solid content of the suspension. Thus, the PVP-to-Ag weight ratio of the Ag NPs could be calculated, shown in Table 1. It is obvious that the PVP-to-Ag weight ratio decreases as the wash times increase. After the fourth washing times, the ratio becomes 0.0490 or the PVP is only 4.9% of the solid Ag NPs. As the amount of PVP approaches a stable value, four times wash is used in this paper to reduce the capping PVP on the surface of Ag NPs. The PVP amount of other Ag NP suspensions (S2 to S4) shows the same decrease tendency as the wash time increases.

**Table. 1:** Analysis data according to TGA curves of Ag NP suspension of S1

Washing times	Weight loss for PVP (300 to 500 °C) (%)	Residual mass of silver (600 °C) (%)	PVP-to-Ag weight ratio
2 times	5.07	92.06	0.0551
3 times	4.81	93.41	0.0515
4 times	4.60	93.92	0.0490
5 times	4.56	93.71	0.0487

#### The Effect of Particle Size of Ag NPs on the Amount of PVP

**Fig. 4:**
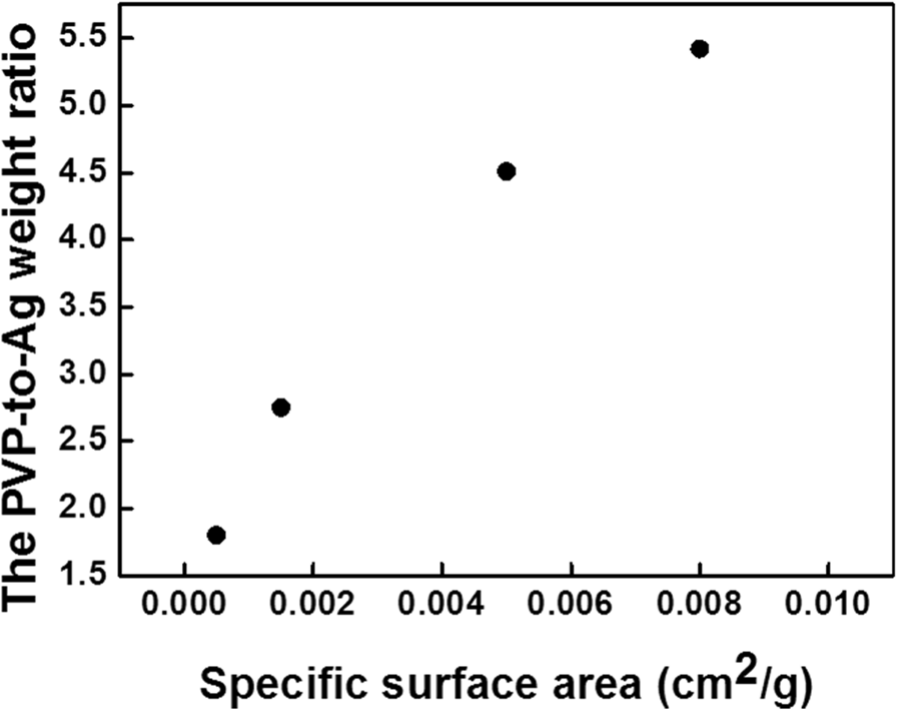
The relationship between the specific area of Ag NPs in different sizes (S1 to S4) and the PVP-to-Ag weight ratio

The Ag NP suspensions of different sizes, S1 through S4, are all being washed for four times to achieved desirable amount of PVP on the surface of Ag NPs (these corresponding TGA curves are shown in Additional file 2: Figure S1.). Using the method mentioned above, the PVP-to-Ag weight ratio for S1 to S4 after four times washing are shown in Table 2. It is obvious that the PVP-to-Ag weight ratio decreases as the average size of Ag NPs increases. Furthermore, the relationship between the specific surface area of the Ag NPs, which was calculated from the particle size and the input amount of precursor, and the PVP-to-Ag weight ratio is shown in Fig. 4. The amount of PVP is likely directly proportional to the specific surface area of Ag NPs. This implies that the protective agent PVP capped on the surface of Ag NPs had similar thickness for each sample or independent of the size of Ag NPs.

**Table 2:** Analysis data according to TGA curves of different sizes of Ag NP suspension (S1 to S4)

Sample	Size (nm)	Weight loss for PVP (300 to 500 °C) (%)	Residual mass of silver (600 °C) (%)	PVP-to-Ag weight ratio
S1	48 ± 12	5.04	92.92	0.0542
S2	76 ± 33	4.26	94.40	0.0451
S3	158 ± 65	2.64	96.04	0.0275
S4	176 ± 85	1.74	96.90	0.0180

### Electrical Resistivity of the Ag NP-Based Film

**Fig. 5:**
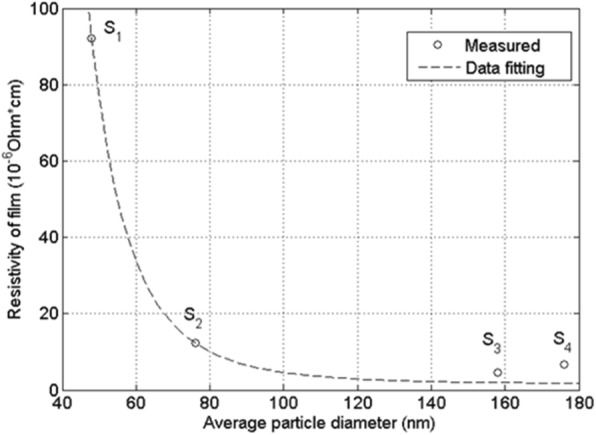
The relationship between the resistivities of the Ag NP-based films and the mean diameter of the Ag NPs at heat temperature of 140 °C. The dashed line is the curve of numerical fitting using Eq. (1)

The electrical resistivity evolution of the Ag NP-based films with respect to particle size (S1 to S4) at various temperatures from 30 to 140 °C for 10 min is shown in Additional file 2: Figure S2. The electrical resistivity of all the four Ag NP-based films decreases as the temperature increases. To further highlight the relationship between the electrical resistivity of the Ag NP-based film and the NP size, the film resistivity at treatment temperature 140 °C for 10 min vs the mean diameter of the Ag NPs is plotted in Fig. 5. As shown, the resistivities of the conductive films decrease monotonically with the particle sizes from 48 ± 12 nm to 158 ± 65 nm. With a smaller size of Ag NPs (48 ± 12 nm), the conductive film exhibited high resistivity, 92.05 μΩ cm. While with a particle size of 158 ± 65 nm, the resistivity decreased to the minimum value of 4.60 μΩ cm, which is only 2.89 times of that of bulk Ag. The resistivity bounced back a little bit when the particle size became even bigger. The explanation why S4 had higher resistivity than S3 is given at the end of this subsection.

For an easier comparison with the existing results, published resistivity values and the corresponding sintering conditions are collected in Table 3. As one can see that the electric resistivities of the Ag NPs obtained in the present work are comparable with those of the reported metallic nanoparticles conductive inks obtained by heat treatment and other kinds of sintering methods including chemical sintering, photonic sintering, IR, plasma, and microwave, considering neither additive agents nor extra equipment was needed in this work, the approach presented in this work is obviously advantageous, which enables to obtain extraordinarily low resistivity at rather low sintering temperature.

**Table 3:** Comparison of sintering condition and electric resistivity of this work with those of in reported literatures

Type of ink	Sintering condition	Electric resistivity (μΩ cm)	Times of the bulk metal resistivity	Ref.
Ag NPs	Chemical sintering at R.T.	7.95	× 5	[﻿20﻿]
Ag NPs	Chemical sintering at R.T.	3.88	× 2.44	[﻿21﻿]
Ag NPs	Chemical sintering at R.T.	9.94	× 6.25	[﻿22﻿]
Ag NPs	Hot pressure sintering 120 °C/25 MPa/15 min	14.3	× 8.99	[﻿23﻿]
Ag NPs	Hot pressure sintering 120 °C/25 MPa/ 20 min	3.92	× 2.47	[﻿24﻿]
Cu NPs	Photonic sintering combining flash light, deep UV and NIR	7.62	× 4.5	[﻿27﻿]
Cu NWs/NPs	Flash light	22.77	× 13.55	[﻿28﻿]
Ag NPs	IR/1 s	10.61	× 6.67	[﻿31﻿]
Ag NPs	IR/15 s	7.95~15.9	× 5~10	[﻿32﻿]
Ag NPs	Plasma	15.9	× 10	[﻿33﻿]
Ag NPs	Plasma combining microwave	2.66	× 1.67	[﻿34﻿]
Reactive Ag ink	120 °C	4.05	× 2.55	[﻿39﻿]
Ag NPs	R.T. for 24 h	~ 8.0	× 5.03	[﻿41﻿]
	140 °C/15 min	~ 5.0	× 3.15	
	180 °C/15 min	~ 3.7	× 2.33	
Ag NPs	140 °C/75 min	10.29	× 6.47	[﻿42﻿]
	160 °C/75 min	3.83	× 2.41	
Ag NPs	60 °C/10 min	13.10	× 8.24	This work
	100 °C/10 min	7.40	× 4.65	
	120 °C/10 min	5.10	× 3.21	
	140 °C/10 min	4.60	× 2.89	

**Fig. 6:**
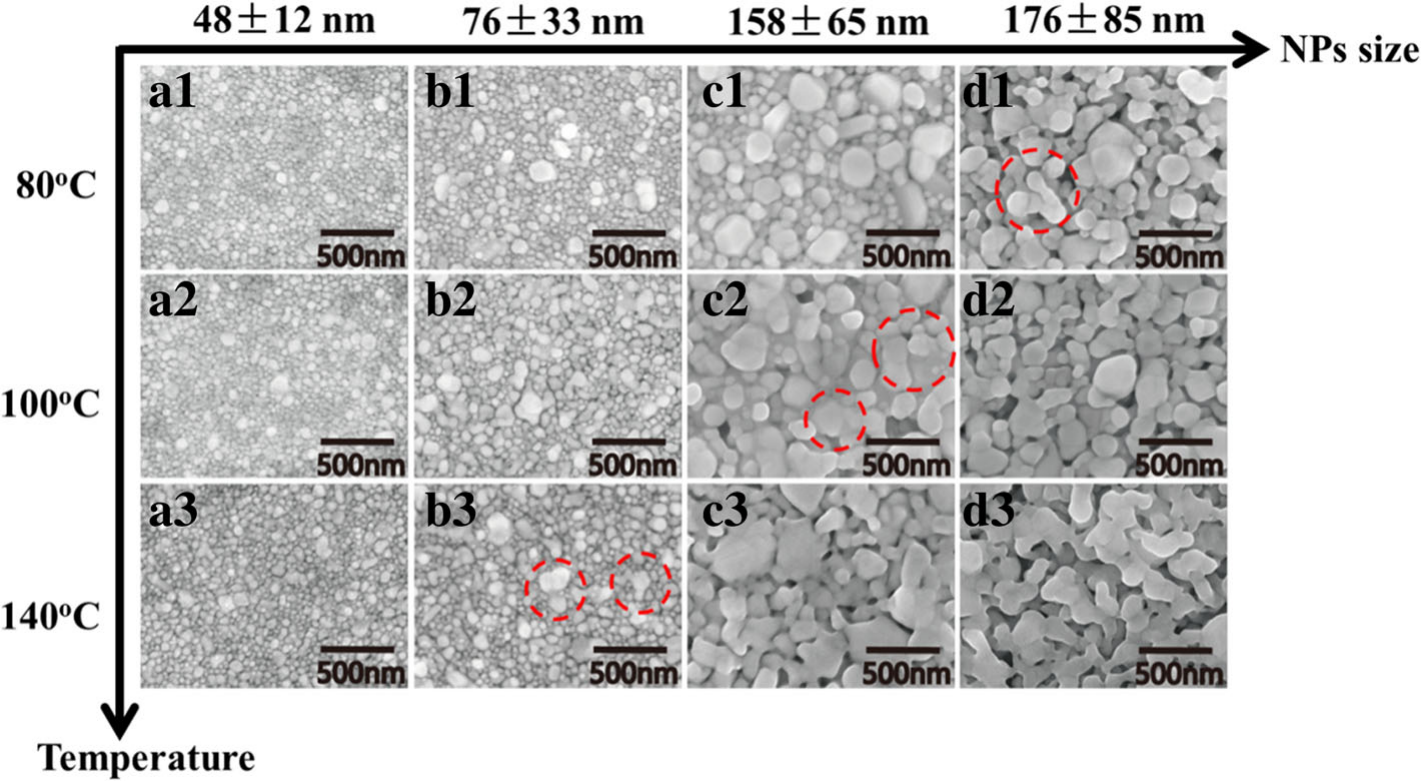
The morphological evolution of the Ag NP-based films at various temperatures with different sizes; the NP size and heating temperature are marked in the coordinate axis

The fact that the resistivity of the conductive film decreased with the increasing size of Ag NPs in the range 48 ± 12 nm to 158 ± 65 nm can probably be attributed to three factors. First, the amounts of PVP capping on the surface of Ag NPs decreased with increasing particle sizes, from 5.42 to 2.75% (see Table 2), which reduced the contact resistance and electron scattering between Ag NPs. Yet, one should be aware of that this reduction is mainly because of reduced surface areas (or specific areas) of the Ag NPs rather than thinner capping thickness of the PVP agent on individual Ag NPs. This is in line with the observation shown in Fig. 3b where the amount of PVP capped on the particle surface was reversely proportional to the size of the obtained Ag NPs. The thickness of the capping layer decreased with the number of washing times and sintering operations. The second is packing density of Ag NPs inside the conductive film. As shown in Table 2, the distributions of the particle sizes are 25, 43, and 41% of the average sizes for S1, S2, and S3 respectively. According to Sohn and Moreland, packing density of a multi particle system increases with extended particle size distribution [40﻿]. A higher packing density may be in favor of improved conductivity in the present study. Third, the relatively deeper sintering level of the bigger Ag NPs compared to that of smaller ones at the same temperature may also be contributed to the decreased resistivity. The detailed investigation was given by the morphology observation of SEM in Fig. 6.

As shown in Fig. 6, with the increasing of the Ag NP size from 48 ± 12 nm to 176 ± 85 nm, the sintering phenomenon of the Ag NP-based film tended to occur at relatively low temperature. For instance, when the Ag NPs of 48 ± 12 nm were chosen, no obvious sintering was observed, and the Ag NPs remained as individuals in the NP-based film at 140 °C (Fig. 6 a3). When the Ag NP size increased to 76 ± 33 nm, the inter-particle necking and initial sintering of the Ag NPs were observed at 140 °C as shown in Fig. 6 b3. We use the dashed-line circles to highlight such phenomenon in the figure. Furthermore, deep level sintering could clearly be observed at 100 °C (Fig. 6 c2) and 80 °C (Fig. 6 d1) for the Ag NP-based film with the sizes of 158 ± 65 nm and 176 ± 85 nm, respectively. As a result, lower resistivity was obtained for the film having relatively larger size of Ag NPs at the same sintering temperature. While this phenomenon seems to be contradictory to the classic theory that the melting point of the metal particles decreases when the size is reduced to the nanoscale [38], it could be attributed to a large amount of PVP capping on the surface of Ag NPs in the case of small particle size, which prevents the inter-particle necking and sintering of the Ag NPs in the film seriously. Therefore, the increase in NP size, the decrease in PVP amount, and the deeper sintering level with dense film morphology, all positivity contributed to a low resistivity of the Ag NP-based film with the NP size ranging from 48 ± 12 nm to 158 ± 65 nm.

The resistivity (6.71 μΩ cm) of the S4 film made of Ag NPs with the average size of 176 ± 85 nm followed the general trend observed from S1, S2, and S3, despite an abnormal increase compared to that of S3 (4.60 μΩ cm). Through careful investigation of the morphology of the Ag NP-based film, we found that the sintered Ag aggregated at 140 °C (Fig. 6 d3) were separated by holes and cracks. This indicates that the further increase in the NP size might have resulted in a certain degree of deterioration in density and conductivity of the Ag NP-based films.

### Relation of the Resistivity with Respect to the Size of Ag NPs

In order to grasp a full picture of how the resistivity of the film changes with the Ag particle size, the measurement values were fitted to the following mathematical expressions:

1 $$ R={R}_0+\frac{C}{r^m} $$

In the expression, *R*_0_ *=* 1.59 is the resistivity of the bulk silver, *r* is the relative particle size normalized to the average particle size of S2 (hence, *r*_2_ = 1), and the constant *C* fulfills the relation, *R*_2_ = *R*_0_ + *C*, where *R*_2_ is the resistivity of S2. The parameter *m* is the fitting parameter determined by fitting to the measured values, i.e., the resistivity values and the average particle diameters of the Ag NPs, S1 through S4.

The underlying considerations of the proposed expression can be summarized into two. First, the resistivity is approaching the intrinsic resistivity of silver bulk when *r* tends to infinity. It is obvious that this constraint is automatically fulfilled by the proposed mathematical expression. Second, the conductivity of the Ag NP films depends solely on the radius of the Ag NPs. The latter can be justified by theoretical reasonings. Provided that the Ag NPs are mono-sized spheres, in addition to the intrinsic resistivity through the silver particles, the resistivity of the film comes mainly from the contact resistance between Ag NPs capped by the PVP protective agent. Hence, one may assume that the resistivity of the film is proportional to PVP-to-Ag weight ratio. The ratio is in turn proportional to the total specific area within a unit volume (unit area of cross section × unit length). As a consequence, we obtain the following relationship,

2 $$ R-{R}_0\propto N\frac{S}{V} $$

where *S* and *V* stand for the surface area and volume of a sphere particle, respectively. Hence,

3 $$ \frac{S}{V}\propto \frac{1}{r} $$

The number of the spherical particle within the unit volume can be estimated by

4 $$ N=\frac{1}{V}\propto \frac{1}{r^3} $$

Thus, in the case of mono-sized spherical particles, the resistivity of the conductive film *R* is

5 $$ R-{R}_0\propto \frac{1}{r^4} $$

Considering that the Ag NPs are neither spherical nor mono-sized, the parameter *m* is thus introduced in the proposed relationship shown in Eq. 1.

Employing the non-linear fitting routine in Matlab and using *R*_0_ *=* 1.59 μΩ cm and the measured value *R*_2_ *=* 12.33 μΩ cm as the inputs, we obtained the parameter, *m* = 4.64. The plot based on the proposed expression is shown in Fig. 5. Obviously, the calculated resistivity values based on the proposed expression are nearly identical to those of S1 and S2 and very close to those of S3 and S4. Considering the broad ranges of the resistivity and particle diameter and there was only one parameter (*m*) involved in the date fitting, the agreement between the calculated values and the measured ones is truly satisfactory.

### Mechanical Flexibility of the Ag NP-Based Ink on Paper

**Fig. 7:**
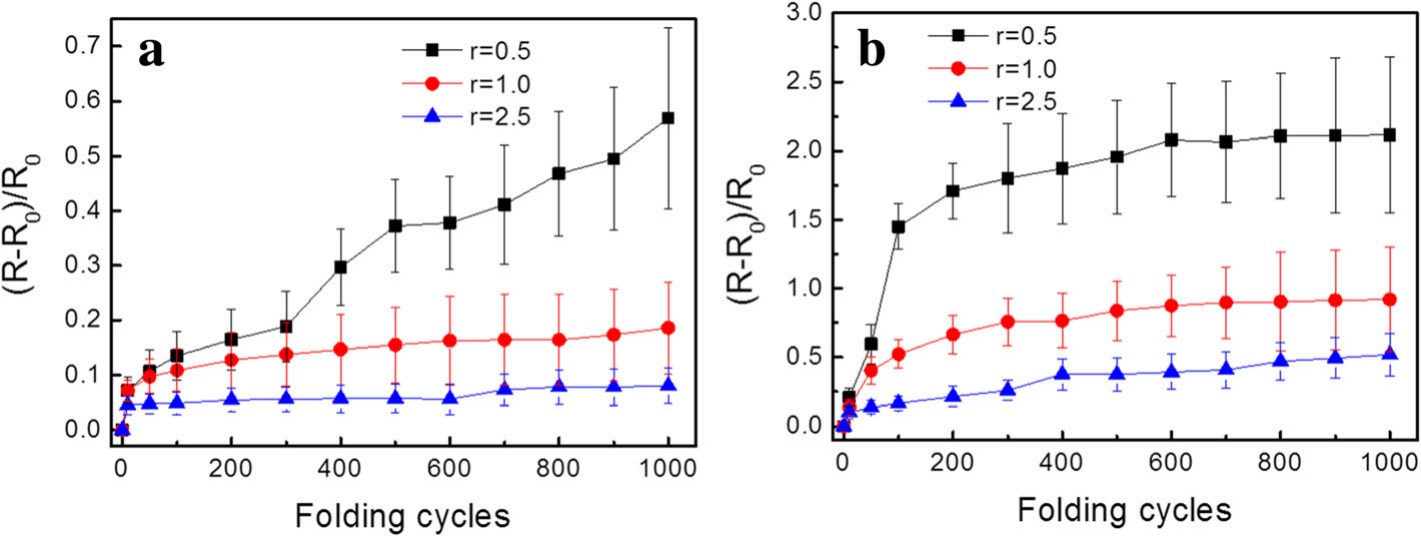
Electrical resistance change rate, (*R* − *R*_0_)/*R*_0_, as a function of bend radius (*r*) and number of bend cycles on **a** art coated paper and **b** photopaper

**Fig. 8:**
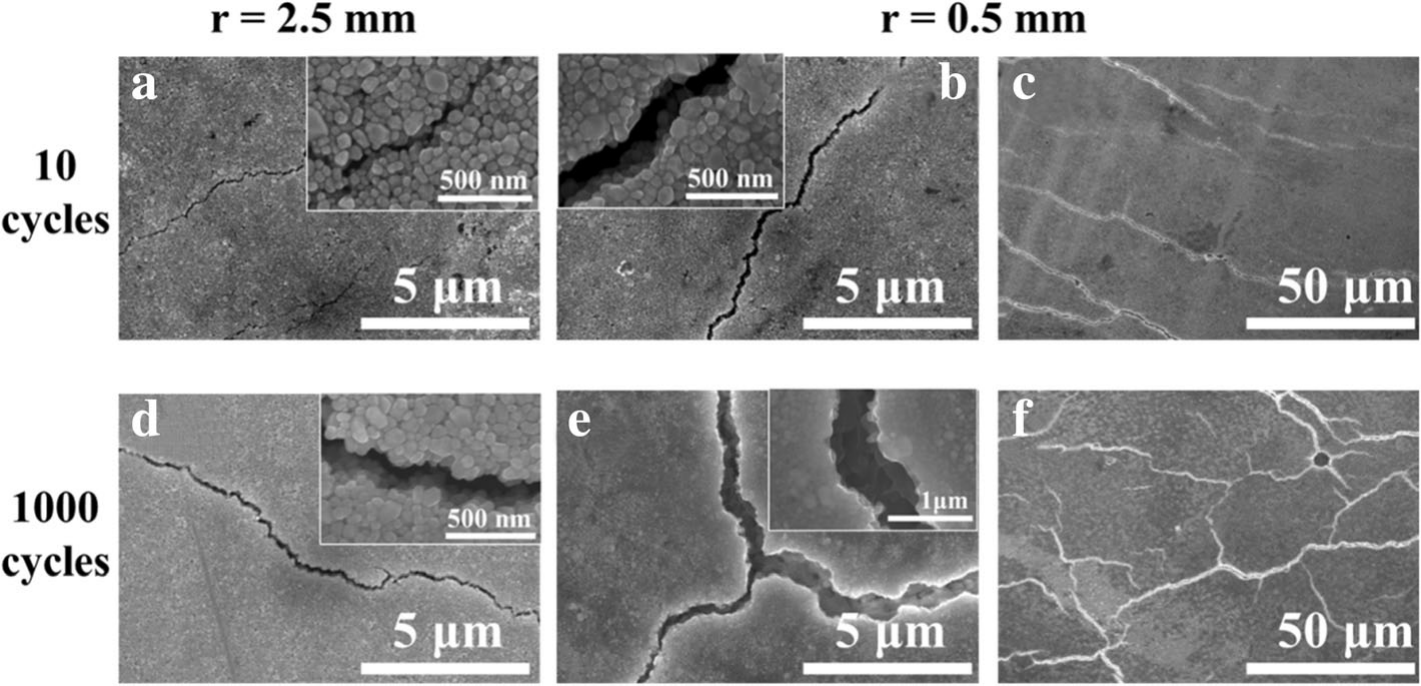
SEM images of the silver electrodes on art coated paper in various bending test conditions. **a** Bending radii of 2.5 mm in 10 cycles. **b**, **c** Bending radii of 0.5 mm in 10 cycles with different magnifications. **d** Bending radii of 2.5 mm in 1000 cycles. **e**, **f** Bending radii of 0.5 mm in 1000 cycles with different magnifications

To investigate the mechanical flexibility of the Ag NP-based ink on paper, bending tests of the printed electronics on art coated paper and photopaper were carried out. Figure 7a shows the bending test results of the silver electrodes on art coated paper. As seen, the samples of bending radii 2.5 mm and 1.0 mm exhibited a robust response over 1000 bending cycles with a slight increase in their electrical resistance. The change rates are 8.01% and 18.55%, respectively. At a closer observation, it was found that such electrical resistance change occurred mainly in the first 10 bending cycles and remained nearly constant in the subsequent testing process. While for the most extreme bend radius of 0.5 mm, the electric resistance evolution of the silver electrodes was rather different. The resistance increases gradually throughout the testing process and increased 56.90% after 1000 bending cycles. To understand the reason of the electric resistance evolution during the bending test, microscopic structure of the silver electrodes on art coated paper was examined by SEM technique. As shown in Fig. 8, cracks with the width of 0.05 μm on Ag NP coating surface were observed after 10 bending cycles in the case of bending radii of 2.5 mm (Fig. 8a). Such cracks remained relatively intact or only slightly propagate into 0.08 μm approximately in the subsequent 1000 bending cycles (Fig. 8d). Consequently, the resistance of the silver electrodes only increased slightly in the very beginning of the bending test then remained constant after that. On the contrary, when tested with a much smaller bend radii 0.5 mm, the cracks on the Ag NP coating surface were up to 0.20 μm wide after 10 initial bending cycles (Fig. 8b). After 1000 bending cycles, the width of the cracks extended to 0.80 μm (Fig. 8e). In the meantime, the orientations of the cracks also changed from initially parallel (Fig. 8c) to all possible directions (Fig. 8f), when the number of bending cycles increased from 10 to 1000. Naturally, the electric resistance of the silver electrodes increased. This suggested that the cracks on Ag NP coating caused by the initial bending cycles could accommodate a large portion of strain applied to the silver array when the bend radius was 2.5 mm or 1.0 mm, resulting in relatively good mechanical flexibility. But when the bending radii decreased to 0.5 mm, the cracks formed in the initial bending cycle cannot accommodate the strain in the following bending cycles resulting in new and larger cracks.

**Fig. 9:**
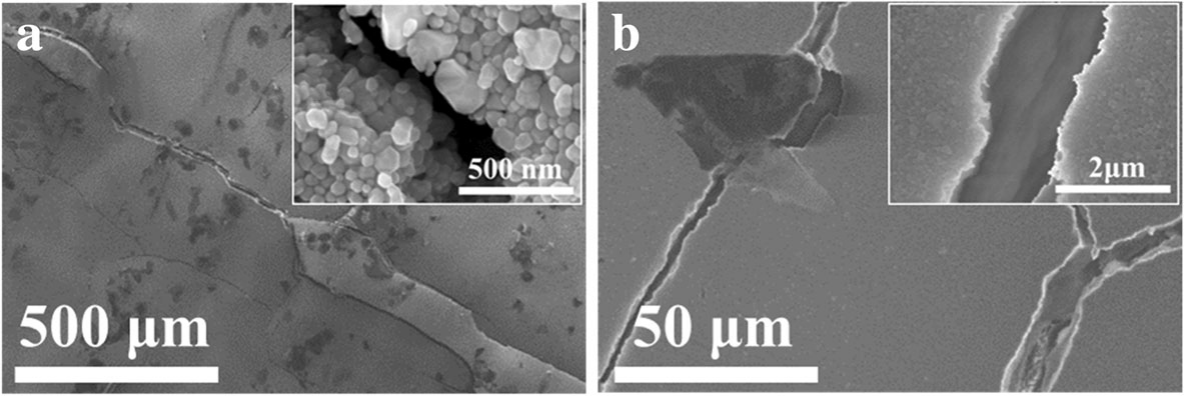
SEM images of the silver electrodes on photopaper with bending radii of 0.5 mm in different cycles. **a** 10 cycles. **b** 100 cycles

For the silver electrodes drawn on photopaper (Fig. 7b), the tendencies of the resistance evolutions were similar to those on the art coated paper when the bending radii were 2.5 mm and 1.0 mm. Yet the resistance stabilized after about 100 initial bending cycles and the corresponding resistance reached a higher level. While for the radii of 0.5 mm, the resistance change rate was even more pronounced. After the initial 100 bending cycles, the resistance increased by 148%. The SEM images shown in Fig. 9 revealed the reason of the sharp increase in the resistance change rate with bending radii of 0.5 mm. As seen from Fig. 9a, cracks of the width of 0.3 μm are clearly observed after only 10 bending cycles. The cracks were deteriorated further when the bending test continued. After 100 initial bending cycles (Fig. 9b), the width of the cracks became 1.8 μm approximately and parts of the Ag NP coating on were even delaminated.

**Fig. 10:**
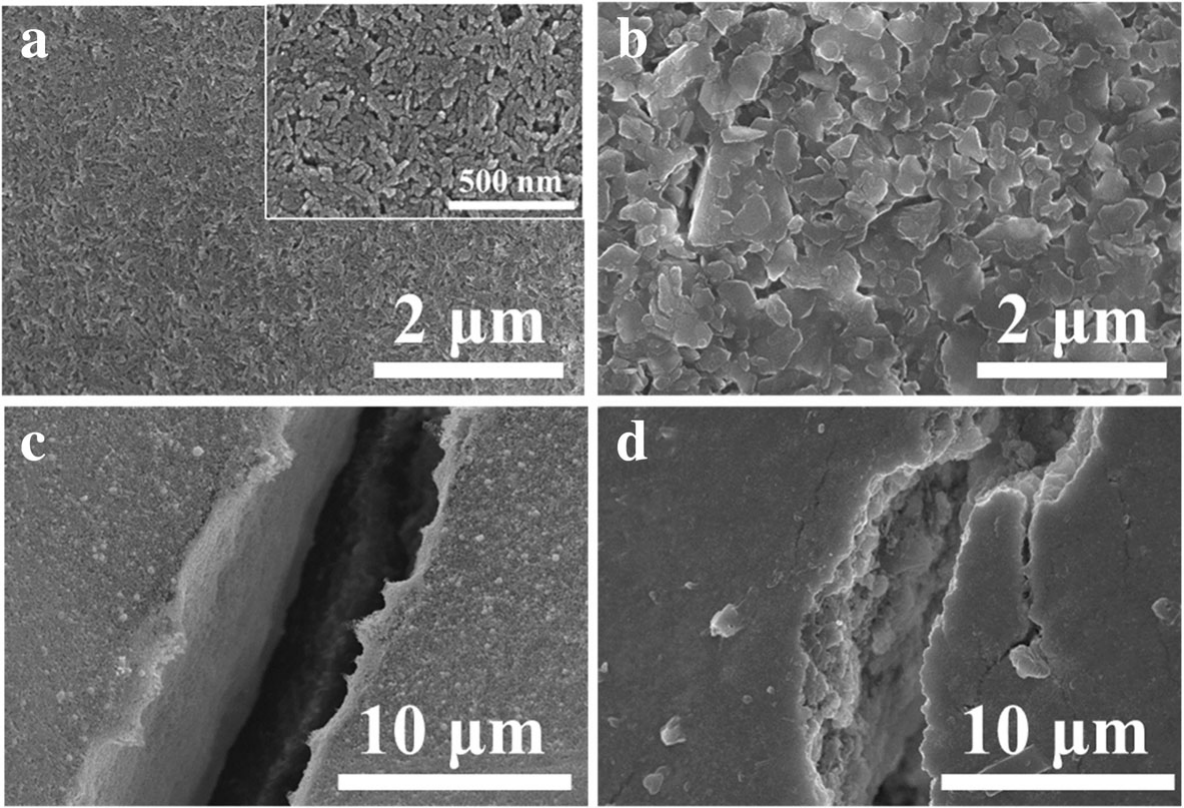
**a**, **b** Surface morphologies of the photopaper and art coated paper. **c**, **d** The microstructure of the Ag NP coating on photopaper and coated paper respectively

The difference in mechanical flexibility between the Ag NP ink patterns might be attributed to the surface morphology of the paper substrates and their corresponding ink absorption property. As shown in Fig. 10a, the surface of the photopaper was made of tightly packed nanoscale particles (probably silica-based) which formed massive nanoscale pores, while the surface of the art coated paper was covered by flake shape coating pigments (probably clay in micron-scale) (Fig. 10b). The observations imply that the art coated paper with coating layer composed of planner and flake-shaped pigments (in micron-scale) may offer better mechanical flexibility compared to that of photopaper. It is well known that ink absorption rate of the substrates (capillary-driven absorption) is inversely proportional to the radii of the pores. Thus, the Ag NP coating on the surface of the photopaper (Fig. 10c) showed an obviously denser microstructure both in the plane and cross section (the crack location is chosen on purpose) compared to that of on the surface of the art paper (Fig. 10d). The dense and compact Ag NP-based coating on the surface of the photopaper might have resulted in a rigid structure, which might also have contributed to the relatively poorer mechanical flexibility compared to that of art coated paper.

### Paper-Based Electronics Applications

To demonstrate the device fabrication capabilities of the low sintering temperature Ag NP-based ink on paper, a 7-segment digital display circuit and a RFID antenna were produced by direct writing and screen printing on the paper substrates, respectively.

**Fig. 11:**
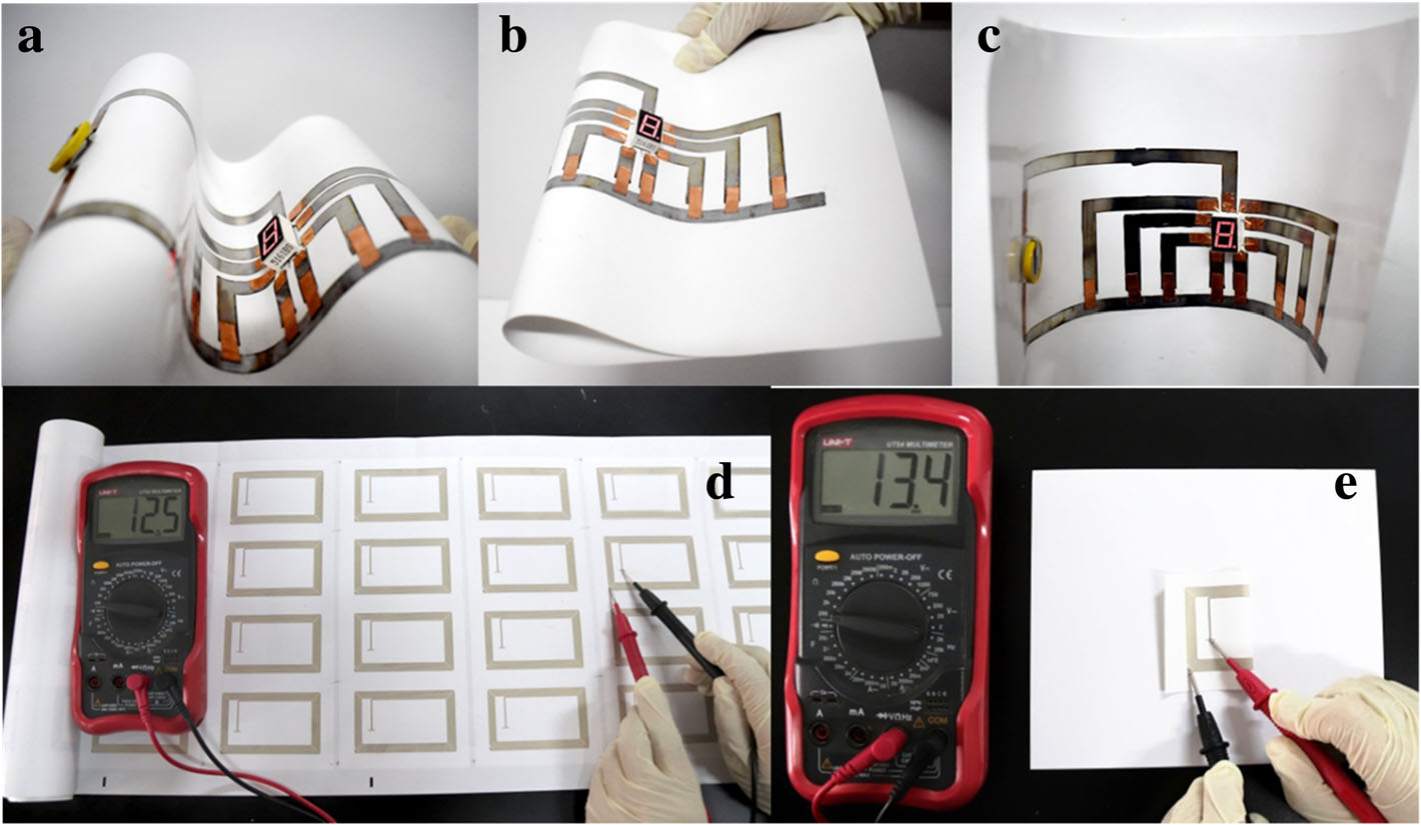
**a**–**c** Hand drawn 7 segment digital LED display circuit bended in various shapes. **d**, **e** Screen printed high-frequency RFID antenna before and after folding

As shown in Fig. 11a to c, a 7-segment digital display circuit was drawn on art coated paper using the Ag NP ink-filled mark pen followed by 120 °C heating for 10 min. Then, a 7-segment LED was surface mounted onto the circuit. To form close electrical contact, the copper foils were used as conductive adhesive to connect the LED and the circuit. We also used copper foils as the switches to control the circuit. The device powered by a 3-V battery worked well when it was bended and crumpled in different shapes, showing excellent mechanical flexibility. A video of the direct drawing 7-segment digital display circuit is shown in the Additional file 1.

The high-frequency RFID antenna was screen printed on art coated paper using the Ag NP-based conductive ink (Fig. 11d). The antenna with the conductive Ag line of 132 cm in length, 1 mm in width, and 7 μm in thickness has a very low resistance of 12.5 Ω after heating at 120 °C for 10 min, which is significantly lower compared to the resistance of the commercial available screen-printed HF RFID antenna of 70 Ω approximately. The printed RFID antenna also shows a good resistance stability changing from 12.5 to 13.4 Ω after face to face folding shown in Fig. 11e.

## Conclusions

High conductive inks demanding for low sintering temperature have been synthesized, using AgNO_3_ and N_2_H_4_·H_2_O as the reactants and PVP as the protective agent. Ag NPs of different size distributions, having the mean radii ranging from 48 to 176 nm, were obtained by adjusting the Ag^+^ concentration in the reaction process. It was observed that the amount of PVP capping agent on the surface of Ag NPs decreased with increasing Ag NP size. There are probably a few factors that influenced the electric resistivity and sintering temperature of the Ag NP-based film. Average size of the Ag NPs is the number one factor affecting the resistivity of the Ag NP film, because the contact resistance amid to interfaces between adjacent Ag NPs played a dominant role. The other factors may be packability of the Ag NPs and the microscopic structure (voids and cracks) of the sintered Ag NP-based film. An empirical expression suggested that the contact resistance decreases with the average radius of the Ag NPs in the form of 1/*r*^4.63^.

The optimal electric resistivity of Ag NP-based film was 4.60 μΩ cm which is only 2.89 times of bulk silver, after 140 °C sintering. This result is generally better than previously reported values obtained with similar sintering method and heating condition. The mechanical flexibility of the Ag NP-based ink on paper substrates was also investigated. The investigation shows that the surface morphology (shape of coating pigments) of the paper substrates and their corresponding ink absorption may be the main factors affecting the mechanical flexibility of the Ag NP conductive ink on the paper substrates. As the demonstrators, two paper-based electric devices were prepared. Their resistances were comparable or eventually better than the commercial product. Thus, the results presented in this study may contribute to the development of low sintering temperature and high conductive inks suitable for paper-based printed electronics.

## Additional files

Additional file 1: A video of direct drawing 7-segment digital display circuit. (MP4 16143 kb)

Additional file 2: **Figure S1.** The TGA curves of the four times washed Ag NP suspensions with different average particle sizes (S1 to S4). **Figure S2.** The electrical resistivity evolution of the Ag NP-based films with different sizes (S1 to S4) during heat treatment. (DOCX 101 kb)

## Abbreviations

EG: Ethylene glycol
NPs: Nanoparticles
PVP: Polyvinyl pyrrolidone
RFID: Radio frequency identification
SEM: Scanning electron microscopy
TGA: Thermogravimetric analysis
