# Supplementary material for: Nano-Silver Ink of High Conductivity and Low Sintering Temperature for Paper Electronics
Source: Nanoscale Res Lett. 2019 Jun 6;14:197. doi: 10.1186/s11671-019-3011-1 (PMC6554461; doi:10.1186/s11671-019-3011-1)
Supplement: Supplementary file 2 — Figure S1. The TGA curves of the four times washed Ag NP suspensions with different average particle sizes (S1 to S4). Figure S2. The electrical resistivity evolution of the Ag NP-based films with different sizes (S1 to S4) during heat treatment. (DOCX 101 kb) [file 11671_2019_3011_MOESM2_ESM.docx]

**Figure S1** The TGA curves of the four times washed Ag NPs suspensions with different average particles sizes (S1 to S4).


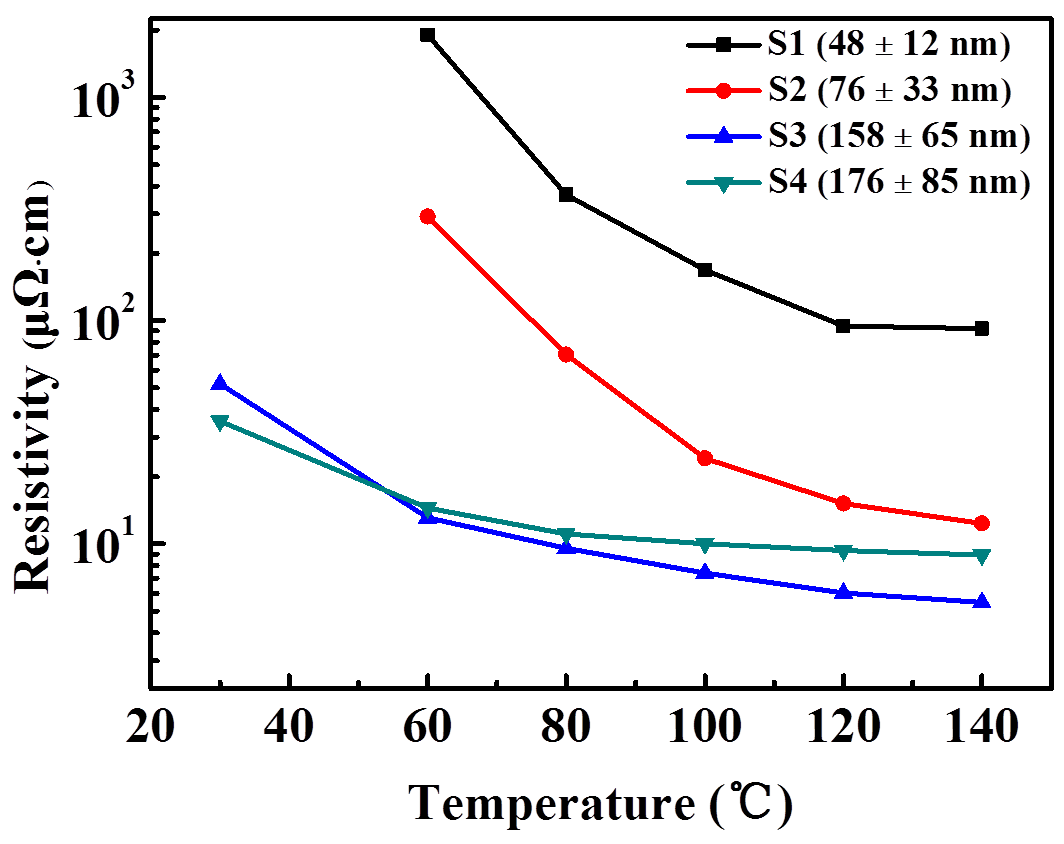


**Figure S2** The electrical resistivity evolution of the Ag NPs based films with different size (S1 to S4) during heat treatment.
